# Web Conversations About Complementary and Alternative Medicines and Cancer: Content and Sentiment Analysis

**DOI:** 10.2196/jmir.5521

**Published:** 2016-06-16

**Authors:** Mauro Mazzocut, Ivana Truccolo, Marialuisa Antonini, Fabio Rinaldi, Paolo Omero, Emanuela Ferrarin, Paolo De Paoli, Carlo Tasso

**Affiliations:** ^1^ CRO Aviano National Cancer Institute Scientific and Patient Library Aviano Italy; ^2^ Infofactory Udine Italy; ^3^ Institute of Computational Linguistics University of Zurich Zurich Switzerland; ^4^ Artificial Intelligence Laboratory Department of Mathematics, Computer and Physical University of Udine Udine Italy; ^5^ CRO Aviano National Cancer Institute Scientific Directorate Aviano Italy

**Keywords:** complementary and alternative medicine, Internet, neoplasms, health information online, website content analysis, barriers to patient-doctor communication, misinformation, sentiment analysis, data mining

## Abstract

**Background:**

The use of complementary and alternative medicine (CAM) among cancer patients is widespread and mostly self-administrated. Today, one of the most relevant topics is the nondisclosure of CAM use to doctors. This general lack of communication exposes patients to dangerous behaviors and to less reliable information channels, such as the Web. The Italian context scarcely differs from this trend. Today, we are able to mine and analyze systematically the unstructured information available in the Web, to get an insight of people’s opinions, beliefs, and rumors concerning health topics.

**Objective:**

Our aim was to analyze Italian Web conversations about CAM, identifying the most relevant Web sources, therapies, and diseases and measure the related sentiment.

**Methods:**

Data have been collected using the Web Intelligence tool ifMONITOR. The workflow consisted of 6 phases: (1) eligibility criteria definition for the ifMONITOR search profile; (2) creation of a CAM terminology database; (3) generic Web search and automatic filtering, the results have been manually revised to refine the search profile, and stored in the ifMONITOR database; (4) automatic classification using the CAM database terms; (5) selection of the final sample and manual sentiment analysis using a 1-5 score range; (6) manual indexing of the Web sources and CAM therapies type retrieved. Descriptive univariate statistics were computed for each item: absolute frequency, percentage, central tendency (mean sentiment score [MSS]), and variability (standard variation σ).

**Results:**

Overall, 212 Web sources, 423 Web documents, and 868 opinions have been retrieved. The overall sentiment measured tends to a good score (3.6 of 5). Quite a high polarization in the opinions of the conversation partaking emerged from standard variation analysis (σ≥1). In total, 126 of 212 (59.4%) Web sources retrieved were nonhealth-related. Facebook (89; 21%) and Yahoo Answers (41; 9.7%) were the most relevant. In total, 94 CAM therapies have been retrieved. Most belong to the “biologically based therapies or nutrition” category: 339 of 868 opinions (39.1%), showing an MSS of 3.9 (σ=0.83). Within nutrition, “diets” collected 154 opinions (18.4%) with an MSS of 3.8 (σ=0.87); “food as CAM” overall collected 112 opinions (12.8%) with a MSS of 4 (σ=0.68). Excluding diets and food, the most discussed CAM therapy is the controversial Italian “Di Bella multitherapy” with 102 opinions (11.8%) with an MSS of 3.4 (σ=1.21). Breast cancer was the most mentioned disease: 81 opinions of 868.

**Conclusions:**

Conversations about CAM and cancer are ubiquitous. There is a great concern about the biologically based therapies, perceived as harmless and useful, under-rating all risks related to dangerous interactions or malnutrition. Our results can be useful to doctors to be aware of the implications of these beliefs for the clinical practice. Web conversation exploitation could be a strategy to gain insights of people’s perspective for other controversial topics.

## Introduction

### Background

Complementary and alternative medicine (CAM) is an umbrella definition for a wide range of medical practices that are no part of the standard medical care [1,2]. These therapies are considered “complementary” if used in addition to a conventional treatment and “alternative” when used instead of it [3].

A survey of the National Institutes of Health reports an increasing trend in CAM usage among American people from 1999-2012 [4]. This trend can be easily compared with similar data that emerged in Europe [5], Asia [6,7], and Africa [8]. The scientific literature about CAM is also growing constantly [5,9-11]. Apart from the research on clinical effectiveness of CAM, many studies on patients’ behaviors and choices are available [11-16]. Today, one of the most relevant topics in literature is the nondisclosure of CAM use among cancer patients [9,13,17].

The Italian context scarcely differs from these trends. A national survey performed by Italian Center for Social Studies and Investments (CENSIS) reports that 23.4% of Italian people resorted to unconventional medicine such as homeopathy and herbal remedies at least once in 2008 [18]. D’Arena [14] reported that CAM use among Italian cancer patients is expanding and mostly self-administered.

Current literature reports that patients would like to talk with their doctors about CAM, but they do not [5,6,9,16]. In particular, they would like to ask for information about safe use of CAM, rather than scientific evidence [16,19]. Today, many studies report that this information need is often unmet [16,17,20,21].

On one hand, usually doctors do not ask patients about their CAM consumption habits [9]. Maybe this is because of doctor’s lack of knowledge of CAM issues [9,15,19,22]. On the other hand, patients anticipate doctors’ disapproval or consider the disclosure irrelevant to their conventional care [9,23]. Consequently, patients autonomously search for information about CAM.

This general lack of communication between doctors and patients about CAM has relevant consequences.

First, it exposes patients to potentially dangerous behaviors [9,14,15,23,24]. Second, health professionals miss relevant information about patients’ needs, beliefs, behaviors, and experiences about CAM therapies [6,13,25-27]. Third, lack of communication eases the spread of misinformation [21,28]. False information has relevant effects not only on patients’ health outcomes [29,30] but also on the decision makers’ policy as well, through collective debates [28,31,32]. Fourth, the lack of communication forces people to use alternative and less-reliable information channels [20,33-36]. Several studies reported that social communities are the main alternative information source about CAM for patients [5,37,38]. Within them, health information flows as a word of mouth mainly driven by narration of people’s experiences, emotions, and opinions [5,28,37,39], in an intense interaction with mainstream media [31,32].

This trend is amplified through the virtual communities that crowd the Web [39,40]. Today, we are able to mine and analyze systematically the amount of unstructured textual information available in websites, forum, social networks, and other digital communities [41]. Some studies extracted relevant information about patients’ point of view about different health topics exploiting social network analysis, natural language processing, content, and opinion mining software [42-44]. This knowledge is implicit in the Web conversations, in the semantic relationships among users, and in their opinions expressed by tags and comments. We hypothesize that the Web is rich of information concerning people’s perspective about CAM topics. Even if expressed in an informal context, Web conversation can give an insight about the untold Italian people opinions, beliefs, and rumors about CAM. This is an implicit knowledge otherwise achievable through the Scientific and Patients Library (SPL) interviews or formal questionnaires.

### Preliminary Data

We collected data about CAM information requests to the SPL of the Centro di Riferimento Oncologico (CRO) Aviano, National Cancer Institute. From 2008 to 2013, 218 of 2313 overall questions requested to SPL (9.4%) were CAM-related. But the trend is constantly increasing. In 2008, we recorded 24 CAM-related questions of 387 (6.2%). In 2012, the CAM-related questions were 38 of 198 (19.2%), and in 2013, 46 of 282 (16.3%). Overall, 183 requests of 218 (84%) regarded biologically based therapies such as diet, food, or natural remedies effectiveness; interactions among remedies and drugs; and remedies availability. We have to point out that these numbers reflect only the questions recorded through the SPL access form. Due to our policy [45], this form is filled by patients to respect their privacy. Consequently, they are not forced to give any information about their requests.

### Aims

Based on the literature and the preliminary data, we aim to analyze Italian Web conversation about CAM. In particular, we aim to identify the most relevant Italian Web conversation sources; the cancer CAM therapies most discussed by Italian Web users and the corresponding sentiment; and what is the most discussed disease.

## Methods

Our data collection methodology could be assimilated to the theoretical sampling [46] because it involved simultaneously collecting, coding, and analyzing textual data from the Web conversations. The textual data analyzed in this work have been collected by means of ifMONITOR [47], an automatic web intelligence tool developed by the Artificial Intelligence Laboratory of the University of Udine. ifMONITOR provided the following services: (1) Web filtering, (2) Web monitoring and thematic database construction, and (3) automatic classification.

**Figure 1:**
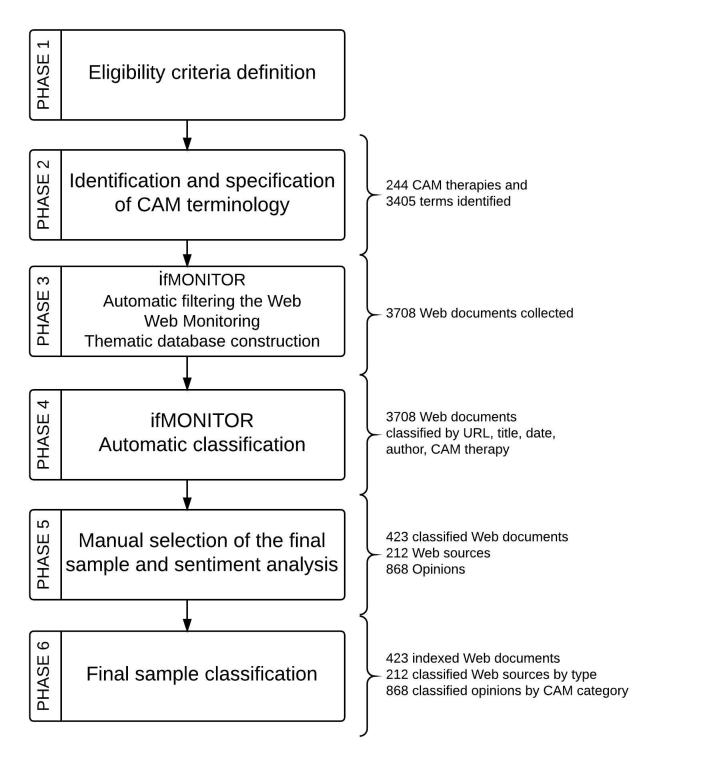
Web conversation analysis flowchart.

Within our search, we followed a systematic approach, which was organized into 6 phases, with a strictly functional approach. We have used ifMONITOR for services “a” and “b” in phase 3 and for service “c” in phase 4. Figure 1 shows the data collection and analysis flowchart. Further details and a flowchart about the complete workflow are available in the Multimedia Appendix 1.

### Phase 1: Eligibility Criteria Definition

Websites, forums, blogs, communities, and social networks of any kind and topic addressed to the general public and containing conversations about CAM topics have been included. In particular, Facebook profiles, pages, and groups of users and YouTube channels were also included (depending on the privacy settings). Otherwise, we decided to leave out Twitter because the length of the messages is too short to express a complex opinion about the topic taken into account. The Web search was also limited to Web documents published between January 1, 2013, and May 31, 2014. We also limited the Web search only to Italian language Web documents, to meet the perspective of Italian people with low literacy skills.

### Phase 2: Identification and Specification of CAM Terminology

**Figure 2:**
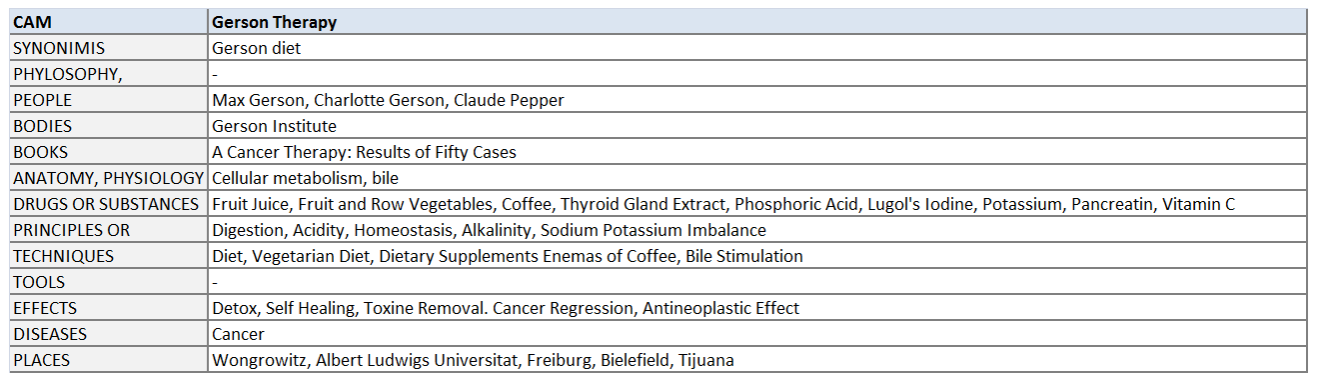
CAM database sample: “Gerson Therapy” item translated into English.

This phase is manual and has been executed by CRO librarians. They built up a CAM database containing relevant terms to be used as a reference in subsequent phases. For each CAM therapy identified, a structured template was filled up with related synonymies and references to specific philosophy or religion; names of people, organizations, and places; book titles; anatomy and physiology terms; substances or drugs; principles or processes; methods; instruments or tools; effects or side effects; diseases. Because the final goal was to analyze Web-based people conversations, it was important to capture the terminology that is actually known by the people themselves. For these reasons, 3 main kinds of sources of information have been considered: (1) scientific and medical resources [1,48,49], (2) specific educational booklets for patients published by cancer volunteer associations [50,51], and (3) public Web-based sources concerning CAM in Italian language easily accessible on the Web [52,53]. Figure 2 shows a sample of our CAM database referring to the “Gerson Therapy”.

### Phase 3: Automatic Filtering of the Web

This phase is aimed at collecting all the materials to be analyzed later exploiting ifMONITOR services “a” and “b.”

The final result of phase 3 has been a set of 3708 documents, largely referring to people’s conversations, which were processed in phase 4.

### Phase 4: Automatic Classification

This fully automated phase is aimed at classifying the 3708 documents retrieved in which CAM therapy is mentioned in the document. The starting point has been the terms included in the CAM therapies database.

### Phase 5: Selection of the Final Sample and Sentiment Analysis

This phase was mainly manual, and it was aimed at identifying a sample of documents to be analyzed in detail. The result constituted 838 Web documents.

The manual analysis of the documents, starting from the documents of the most successful Web-based sources (websites, blog, and forums that published a high number of pages concerning CAM therapies). These criteria enabled us to consider the most popular sources and Web conversations first.

This process continued up to the accumulation of 423 Web documents, when we reached the saturation of the sample [46]. We considered this a significant and feasible size for the sample of documents to be manually analyzed in detail because the rest of the Web documents retrieved did not shed any further light to the evaluation on the most relevant Web conversation sources or the most mentioned CAM therapies.

For each of the 423 documents, manual tagging was added, concerning the sentiment about the perceived effectiveness or ineffectiveness of the CAM therapy mentioned through a numerical score ranging from 1 to 5 (1 very bad; 2 bad; 3 neutral; 4 good; and 5 very good). The score assignment was performed by a team of 4 trained Web intelligence analysts. They performed individually the sentiment analysis on different sets of documents, later they compared together the score assignment, thus limiting information bias due to interoperator discretionary.

**Figure 3:**
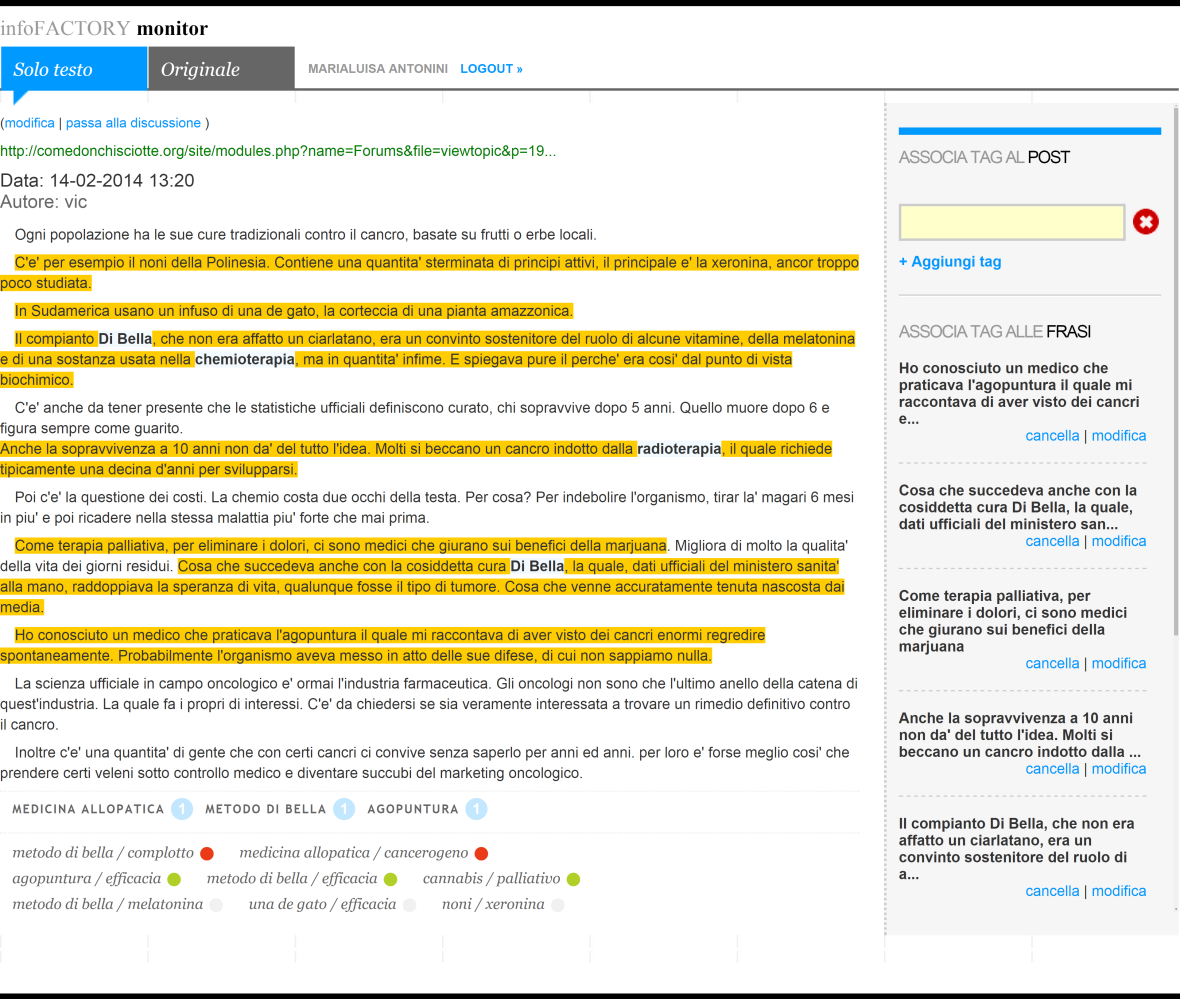
ifMONITOR thematic database screenshot. Highlighted in yellow are the opinions stated in the Web document. In capitals is the number of relevant occurrences automatically indexed by ifMONITOR: allopathic medicine (chemotherapy and radiotherapy); Di Bella Multitherapy, acupuncture. In italics followed by a red, green, or gray spot, the sentiment analysis about the issues stated in the document. Red spot (bad sentiment): plot against Di Bella Multitherapy; allopathic medicine carcinogenic effects. Green spot (good sentiment): acupuncture effectiveness; Di Bella Multitherapy effectiveness; Cannabis as palliative care effectiveness. Gray spot (neutral sentiment): melatonin in Di Bella Multitherapy; Uma de Gato effectiveness; noni and xeronina effectiveness.

We did not perform any assessment of the medical and scientific reliability of these statements. Figure 3 shows a screenshot of ifMONITOR thematic database.

### Phase 6: Final Sample Classification

According to the literature [1,5,24,49], the librarians tagged the documents included in the final sample using five CAM therapies classes. Within each class, they grouped CAM therapies into 14 categories as shown in Table 1.

Furthermore, librarians indexed the Web sources according to the criteria of health and nonhealth thematic areas. Web sources are websites, blogs, forums, and social networks (personal profiles, groups of users, or public pages). Then they gathered Web sources according to their specific subject matters, as shown in Table 2.

The content classification allowed us to identify the most discussed CAM therapies; the most relevant Web sources; and the most discussed cancer type. Furthermore, the semantic analysis allowed us to measure the popularity and sentiment about the identified CAM and identify the pros and cons issues in the discussions about the most discussed CAM therapies.

**Statistical Analysis**

Descriptive statistical analysis was performed using Microsoft Excel. Absolute (n) and relative frequencies (%) with reference to total Web sources, total Web documents, and/or total opinions included in the final sample were computed. For each item expressed using a numerical score (Likert-type scale ranging from 1=very bad to 5=very good) mean scores (mean sentiment score [MSS]) as a central tendency index and standard deviations from the mean (σ) as an indicator of variability were also calculated.

**Table 1:** CAM therapies.

CAM category		Description
Biologically-based		
	Nutrition	Food or diets (ie, veganism, Gerson diet, garlic, green tea)
	Chemical-based medicines	Drugs or remedies based on chemical substances (ie, UK101, baking soda)
	Plant-based medicines	Drugs or remedies derived from plants (ie, Aloe, Essiac, Laetrile)
	Animal-based medicines	Drugs or treatments derived from animals (ie, Escozul, Shark fin)
	Mineral-based medicines	Drugs or treatments derived from minerals (ie, Alcaline water)
Whole medical systems		
	Alternative medical systems	Medical systems apart from standard medicines (ie, Olism, New German Medicine, Omeopathy)
	Traditional medical systems	Remedies and treatments based on folk traditions (ie, traditional Chinese medicine, Ayurvedic medicine)
Mind body interventions		
	Meditation and relaxation	Techniques exploiting the mind's ability to affect biological functions (ie, mindfulness, yoga, hypnosis)
	Psychological approaches	Psychological approaches or stimulation to release the stress (ie, Simonton method, music therapy, color therapy)
	Spirituality	Techniques based on supernatural or divine intervention (ie, pray, self-healing, faith)
Energy therapies		
	Natural energies	Techniques based on natural energies such as heat (ie, hyperthermia, bioenergy, hydrotherapy)
	Spiritual energies	Techniques based on supernatural energies (ie, pranotherapy, Reiki)
Manipulative and body-based		
	Exercises	Techniques based on physical exercises (ie, dance therapy, Tai Chi, silvotherapy)
	Massages	Techniques based on body manipulations (ie, reflexology, osteopaty, chiropratic)

**Table 2:** Web sources classification

Web sources category		Description
Health-related		
	CAM	Fostering of CAM therapies, including cancer CAM therapies
	Health and wellness	Health communication and healthy lifestyles promotion
	Oncology	Cancer patients communities
Nonhealth-related		
	Mainstream newspaper on the Web	Newspaper, both national or local, print or Web-based
	Pseudoscience	Fostering of pseudoscientific or plot theories
	Debunking	Exposition of Web-based pseudoscientific theories
	Other	Any other issues retrieved

## Results

### Phase 2: Identification of CAM Terminology

The CAM Therapy Database included a set of 224 CAM therapies: 19 cancer treatments (ie, Essiac, Gerson Therapy, Escoazul); 205 general remedies used to manage diseases symptoms, treatments side effects, or to cope with the stress (ie, meditation). The number of terms collected referring to the 224 CAM therapies is 3405.

Phase 3 to 6: From Classification to Sentiment Analysis

#### Web Documents Overall

The final sample included 423 Web documents extracted from 212 Web sources, showing 868 opinions.

Ten Web sources including 39 Web documents tagged automatically by ifMONITOR have become inaccessible before Phase 6. Nevertheless, all of them were automatically indexed by URL, title, author, date, CAM therapy mentioned, and opinion. But they lack information about Web sources or Web documents subjects (for instance topic of the single Web document). The 868 opinions retrieved stated an overall MSS of 3.6 (σ=1.0).

We found 244 of 868 (28.1%) opinions related to a specific disease: 81 of 244 (33.2%) were breast cancer related; 64 (26.2%) were pancreatic cancer related; 29 (11.9%) were melanoma related; 27 (11.1%) were lung cancer related; 21 (8.6%) were bone marrow cancer related; 14 (5.7%) were ovarian cancer related; 6 (2.5%) were peritoneum cancer related; 1 (0.4%) were neck cancer; and 1 (0.4%) were mandible cancer related.

#### Web Sources

We found 95 of 212 websites (44.8%), 45 social networks (21.3%), 41 blog (19.3%), and 28 forums (13.2%).

Within social networks, 22 of 45 (49%) were public personal profiles, 12 (27%) were groups of users, and 11 (24%) were company or personal pages.

We found 83 of 212 (39.2%) “health-related” Web sources producing 176 of 423 (41.6%) Web documents. Among the “health-related” group, we found 46 of 212 (21.7%) Web sources focused on CAM topics producing 103 of 423 (24.3%) Web documents. The 377 opinions of 868 (43.4%) of this group, expressed a MSS of 3.7 (σ=0.8). The Web sources focused on “oncology” topics were 8 of 212 (3.8%), producing 37 of 423 (8.7%) Web documents.

On the other hand, 126 of 212 (59.4%) were “nonhealth-related” Web sources, producing 237 of 423 (56%) Web documents. Among “nonhealth-related” group, we found 66 of 212 (31.1%) Web sources focused on a wide range of different topics that produced 142 of 423 Web documents (33.6%). The 476 opinions of 868 (54.8%) retrieved into this group, expressed a MSS of 3.6 (σ=1.1).

The highest MSS (3.7) was observed among “other issues,” “oncology,” “health and wellness,” and “CAM” categories. The lower (2.7) within the “debunking” category. Complete results are displayed in Table 3.

**Table 3:** Results per Web source.

Web Sources category		WS^a^, n (%)	WD^b^, n (%)	OP^c^, n (%)	MSS^d^ (±σ^e^).
Health-related		83 (39.2)	176 (41.6)	377 (43.4)	3.7 (± 0.8)
	CAM	46 (21.7)	103 (24.3)	198 (22.8)	3.7 (±0.8)
	Health and wellness	29 (13.7)	36 (8.5)	79 (9.1)	3.7 (±0.1)
	Oncology	8 (3.8)	37 (8.7) 100 (11.5)	3.7 (±1.1)	
Nonhealth-related		126 (59.4)	237 (56)	476 (54.8)	3.6 (±1.1)
	Mainstream newspaper on the Web	26 (12.3)	35 (8.3)	93 (10.7)	3.4 (±1.1)
	Pseudoscience	30 (14.2)	52 (12.3)	124 (14.3)	3.6 (±1.1)
	Debunking	4 (1.9)	8 (1.9)	13 (1.5)	2.7 (±1.2)
	Other	66 (31.1)	142 (33.6)	246 (28.3)	3.7 (±1.1)
Unknown		3 (1.4)	10 (2.4)	15 (1.7)	3.9 (±0.3)
					
Total		212 (100)	423 (100)	868 (100)	3.6 (±1.0)
					

^a^WS: Web sources.

^b^WD: Web documents.

^c^OP: opinions.

^d^MSS: mean sentiment score.

^e^σ: standard deviation.

Yahoo Answers is the most retrieved Web source: 41 of 423 (9.7%). The Facebook group “Quelli che il cancro (e non solo) lo curo a modo mio” (those who cure cancer, and not only, in their own way), counted 30 Web documents of 423 (7.1%). This group changed its privacy settings: today the access is limited to the registered members only. But if we sum all Facebook profiles, groups, and pages together, we count 89 of 423 (21%) Web documents, nearly double that of Yahoo Answers. The third Web source per number, 14 of 423 (3.3%), was Greenstyle.it, a Web magazine fostering ecologic lifestyles owned by HTML.it, an Italian network of publishing and advertising companies.

#### CAM Therapies

We found 94 therapies or remedies mentioned in the conversations retrieved. Among these, 68 (72%) were included in the CAM keyword set. Twenty-six (28%) are therapies that emerged during the manual reviews of ifMONITOR’s search output in phase 3. Some of these are typical Italian alternative cancer remedies, such as “Giuseppe Nacci’s” diet, an Italian variation of “Gerson Therapy,” “Gianfranco Pantellini’s” therapy, based on potassium ascorbate consumption, “Alessiani’s Water,” enriched with minerals extracted from Romans catacombs.

The most discussed CAM therapies belong to the “biologically based therapies” group, with 702 of 868 (80.9%) opinions retrieved. The MSS observed was 3.6 (σ=1.0). Among “biologically based therapies” the “nutrition” category had the higher rate of opinions, 339 of 868 (39.1%), expressing an MSS of 3.9 (σ=0.8). “Chemical-based medicines,” counted 186 opinions of 868 (21.4%) showing an MSS of 3.3 (σ=1.3). “Plant-based medicines” counted 144 opinions of 868 (16.6%), and an MSS of 3.6 (σ=1.0). The complete results are displayed in Table 4.

**Table 4:** CAM therapies per category.

CAM category		OP^a^, n (%)	MSS^b^, (±σ^c^)
Biologically-based therapies		702 (80.9)	3.6 (±1.0)
	Nutrition	339 (39.1)	3.9 (±0.8)
	Chemical-based medicines	186 (21.4)	3.3 (±1.3)
	Plant-based medicines	144 (16.6) 3.6 (±1.0)	
	Animal-based medicines	26 (3.0)	3.5 (±0.6)
	Mineral-based medicines	7 (0.8)	4.0 (±0.6)
			
Whole medical systems		103 (11.9)	3.5 (±1.0)
	Alternative medical systems	81 (9.3)	3.4 (±1.1)
	Traditional medical systems	22 (2.5)	4.0 (±0.8)
			
Mind–body interventions		32 (3.7)	4.0 (±0.6)
	Meditation and relax	20 (2.3)	4.1 (±0.7)
	Psychological approaches	6 (0.7)	4.2 (±0.4)
	Spirituality	6 (0.7)	3.8 (±0.4)
			
Energy therapies		20 (2.3)	3.9 (±0.7)
	Natural energies	15 (1.7)	4.0 (±0.6)
Spiritual energies	5 (0.6)	3.4 (±0.9)	
			
Manipulative and body-based		11 (1.3)	3.8 (±0.4)
	Exercises	8 (0.9)	3.9 (±0.3)
	Massage techniques	3 (0.3)	3.7 (±0.6)
			
Total		868 (100)	3.6 (±1.0)
			

^a^OP: opinions.

^b^MSS: mean sentiment score.

^c^σ: standard deviation.

We identified different approaches concerning the “nutrition” issues. On one hand, most of the time, Web users talked about the effectiveness of a certain diet or alimentary regimen in cancer treatment or prevention. For instance, “vegetarianism” or “veganism,” “raw foodism,” “alkaline diet,” “Gerson therapy.” On the other hand, they often discussed about the healing properties of a specific food or spice. For instance, grapefruit, garlic, turmeric, mushrooms, lemon, or green tea.

Finally, the discussions on dietary regimen included also the use of supplements, for instance, vitamins. “Diets” overall collected 154 opinions of 868 (18.4%) with an MSS of 3.8 (σ=0.9). “Food as CAM” overall collected 112 opinions of 868 (12.8%) with a MSS of 4 (σ=0.7). “Supplements” collected 73 opinions of 868 (7.8%) with an MSS of 3.8 (σ=0.9). The main results are summarized in Table 5.

Excluding diets and food, the most discussed CAM therapy is the “Di Bella multitherapy.” We retrieved 102 opinions of 868 (11.8%) with an MSS of 3.4 (σ=1.2). “Di Bella multitherapy” and a reportage of “Le Iene,”(the Hyenas) Italian comedy and satirical TV show with reports on politics and consumers issues, on “alkaline diet” and “veganism” were also the most recurring topic: 16 of 423 (3.8%) Web documents retrieved.

**Table 5:** Most discussed CAM therapies.

CAM classification		CAM category	OP^a^, n (%)	MSS^b^, (±σ^c^)
Diets				
	Biologically-based therapies Nutrition	154 (18.4)	3.8 (±0.9)	
Food as CAM				
	Biologically-based therapies	Nutrition	112 (12.8)	4.0 (±0.7)
Di Bella multitherapy				
	Biologically-based therapies	Chemical-based medicines	102 (11.8)	3.4 (±1.2)
Phytotherapy				
	Biologically-based therapies	Plant-based medicines	96 (11.1)	3.6 (±1.0)
Supplements				
	Biologically-based therapies	Nutrition	73 (7.8)	3.8 (±0.9)
Simoncini therapy				
	Biologically-based therapies	Chemical-based medicines	45 (5.2)	2.6 (±1.4)
Naturopathy				
Whole medical systems	Alternative medical systems	38 (3.7)	3.5 (±1.0)	
Father Zago’s aloe remedy				
	Biologically-based therapies	Plant-based medicines	22 (2.5)	3.6 (±0.9)
Others				
		-	226 (26)	-

^a^OP: opinions.

^b^MSS: mean sentiment score.

^c^σ: standard deviation.

#### The Case of Di Bella Multitherapy

We found 29 of 212 (13.7%) Web sources containing 58 of 423 (13.7%) Web documents and 102 of 868 (11.7%) opinions about “Di Bella multitherapy.” This is a controversial cancer treatment invented by the Italian physician Luigi Di Bella, based on a mix of somatostatin, melatonin, hormones, and vitamins. In the early 1990s, this treatment had a wide appeal among Italian cancer patients [54]. The trial performed in 1998 by the Italian Ministry of Health proved that this therapy is ineffective [54-58]; nevertheless, it has been followed by an intense emotional campaign in favor of the therapy [54]. For these reasons, patients rarely ask for information or disclose the use of Di Bella multitherapy to their doctors. Three Web sources containing 8 Web documents were not accessible in phase 6. The complete results are displayed in Table 6.

**Table 6:** Discussions about Di Bella multitherapy.

CAM category		WS^a^ , n (%)	WD^b^, n (%)	OP^c^, n (%)	MSS^d^, (σ^e^)
Health-related		8 (28)	15 (26)	37 (36.3)	3.4 (±1.1)
	CAM	3 (10)	4 (7)	8 (7.9)	3.0 (±1.1)
	Health and wellness	0 (0)	0 (0)	0 (0)	-
	Oncology	5 (17)	11 (19)	29 (28.4)	3.6 (±1.1)
Nonhealth-related		21 (72)	43 (74)	65 (63.7)	3.3 (±1.3)
	Mainstream newspaper on the Web	6 (21)	9 (15)	13 (12.7)	3.5 (±1.2)
	Pseudoscience	6 (21)	9 (15)	17 (16.6)	3.8 (±1.1)
	Debunking	0 (0)	0 (0)	0 (0)	-
	Other	9 (31)	25 (43)	35 (34.4)	3.1 (±1.3)
Total		29 (100)	58 (100)	102 (100)	3.4 (±1.2)

^a^WS: Web sources.

^b^WD: Web documents.

^c^OP: opinions.

^d^MSS: mean sentiment score.

^e^σ: standard deviation.

This therapy was mostly discussed among “Nonhealth-related” web sources, with 65 out of 102 (63.7%) opinions retrieved showing an MSS of 3.4 (σ=1.1). Most of the conversation were retrieved among the “other issues” category, with 35 of 102 (34.4%) opinions showing an MSS of 3.1 (σ=1.1). No opinions were retrieved into the “debunking” and “health and wellness” category.

Conversely, we counted 37 of 102 (36.3%) opinions into “health-related” Web sources showing an overall MSS of 3.3 (σ=0.8). We observed that it was discussed almost only in the “oncology” category, with 29 of 102 (28.4%) opinions retrieved showing an MSS of 3.6 (σ=1.1).

We also found that 23 of 423 (5.4%) Web documents have the “Di Bella multitherapy” as main topic of the conversation, distributed among: 6 in “oncology,” 1 in “CAM,” 2 in “mainstream newspaper on the Web,” 2 in “pseudoscience and conspiracy theories,” and 12 in “other issues” categories.

The conversations retrieved were mainly focused on the discussion about effectiveness or ineffectiveness of “Di Bella therapy,” with 60 of 102 (58.8%) opinions showing an MSS of 3.8 (σ=1.3). The lowest MSS observed is 2.5 (σ=1.1) achieved by the topic “plot against therapy’s trial” means that the 5 users believe to the plot theory against the “Di Bella multitherapy.”

The arguments in support of the Di Bella multitherapy expressed by users in the Web conversations were the absence of side effects, the effectiveness in treating neoplasm otherwise terminal, and its effectiveness while conventional therapies are suspended. There is a widespread belief that its real effectiveness cannot be proven because the trial was intentionally compromised. To prove this last statement, users often report that the well-known oncologist Umberto Veronesi during an interview confirmed the effectiveness of the somatostatin in breast cancer treatment. The points against the Di Bella multitherapy were mainly two: the therapy has not been recognized as a standard therapy by the Italian National Health Service because of the lack of scientific evidence and the therapy has high initial costs.

## Discussion

### Principal Findings

Our survey confirms the existence of Web-based conversations about cancer CAM therapies among Italian Web users. The overall sentiment on CAM effectiveness tends to range from neutral to good, with a certain degree of variability in opinions (MSS±: 3.6±1.0).The conversations about CAM were retrieved mostly from “nonhealth-related” rather than “health-related” web sources. Facebook and Yahoo Answers together covered almost one third of the conversation retrieved. Beyond them, most of the CAM conversations are widespread within Web sources that deal with very different topics. For instance, political activism, economy, marketing, video games, sport, gastronomy, leisure, and weather forecasts. The overall sentiment observed in the “nonhealth-related” category is quite neutral with high level of polarization (MSS±: 3.3±1.3).

A similar overall sentiment emerges in the “health-related” category, although with less variability (MSS±: 3.4±1.1). Despite the low number of Web sources retrieved, we found a high rate of opinions in the “oncology” category. This is because they are mostly conversations among patient-dedicated forums users. The sentiment observed in this category is quite good (MSS=3.8), with some variability among the conversations partaking (σ=1.1).

The most discussed CAM therapies belong largely to the “biologically-based” category. In particular, the “nutrition” has a good score with low polarization (MSS±: 3.9±0.8). “Nutritional” CAMs are also considered very effective for cancer prevention, side-effects management, and as cancer cure also. Nutritional conversations concern the effectiveness of diets, such as “veganism.” But they also concern the healing properties of a single food. In this case, the consensus is high and with quite low variation (MSS±: 44±0.9). “Plant-based” medicines are also perceived as quite effective (MSS±: 3.6±1.0). Against the number of opinion retrieved, we observed an almost neutral sentiment score with a quite high polarization of opinions (MSS±: 3.3±1.3) among “chemical-based” medicines.

Only a third (28.1%) of the opinions retrieved mentioned a specific neoplasm: mostly breast or ovarian cancer. In the other case, people referred only to cancer in general.

Contrary to what we expected, we collected a low number of very popular CAM, such as homeopathy, Chinese traditional medicine, acupuncture, yoga, and reflexology. At the same time, almost a third (28%) of the CAMs that emerged during the manual reviews in phase 3 were unexpected or uncommon. They are all “biologically based.”

Finally, particularly significant is the finding of a considerable amount of opinions about the “Di Bella multitherapy.” We observed that Di Bella multitherapy is mentioned mostly within the “nonhealth-related” Web sources. The corresponding MSS tends to a neutral value, with a very high rate of polarization, especially among the “nonhealth-related” Web sources (σ 1.3). It is interesting to observe that the higher MSS is recorded among the “oncology” and “pseudoscience and conspiracy theories” categories together.

These findings on Di Bella Multitherapy are particularly relevant if we compare its popularity with other similar pseudoscientific cancer treatments, such as the “Simoncini Baking Soda” therapy. This latter is notably less discussed, with a very low MSS and a very high level of polarization (σ 1.4). This proves that Di Bella multitherapy is considered more than a pseudocure by those partaking in the Web conversations that were considered.

### Comparison With Prior Works

Our findings in number and distributions of conversations confirm those reported in the Social Oncology Project for the United States [39]: cancer conversations are ubiquitous. The sentiment expressed in the considered Italian Web conversations are comparable with those observed in Israel [6] about the perception of effectiveness and safety of CAM (mean scores were equal to 3.4619 and 3.6589, respectively, using a similar 1-5 score range).

The distribution of the conversations complies with the results of the CENSIS national report [38]: only 13.9% of the Italian people that are used to share advices and experiences in forum or specific Web-based communities. Conversely, almost 90% of Italian people exploit search engines to find the health information they need. Very generic Web sources such as Facebook and Yahoo Answers are the main conversation triggers. Facebook users seem to be more active in promoting diets and healthy lifestyles (ie, naturopathy and ecology). But we also found several pages that promoted pseudomedicine practitioners or products (ie, Escozul). Yahoo Answers users behavior differ if they are “askers” or “answerers.” The askers show a rather low knowledge about cancer topics and about the differences among its typologies. They ask very generic questions such as: “What is the name of the disease that make you go bald?” or “What is the cancer cure?” or “Is there a cure for cancer alternative to chemotherapy?” The answerers are also very generic in their replies. But we found that the most active answerers are strong promoters of alternative remedies rather than proper informers (ie, the motto “Di Bella multitherapy” used as profile’s avatar).

This complies with Quattrociocchi [32] and Bessi [28], who observed that in science/pseudoscience Facebook conversations consumers of alternative news are more focused on their contents, whereas scientific news consumers are more prone to comment on alternative news. Moreover, Mocanu [31] observes that those with strong preferences for alternative information sources, perhaps motivated by the will to avoid the manipulation employed by mainstream media controlled by the government are more susceptible to false information. According to Del Vicario [59], Facebook users tend to select and share content related to specific narratives and ignore the rest. This way, conversations often occur in “echo chambers” which cause reinforcement of confirmation bias, shaping users’ commitment to a specific system of beliefs and fostering the spread of misinformation. Particularly, Bessi [28] observed also that unsubstantiated scientific claims and rumors reverberate over a period of time comparable with that of more verified information among the Italian Facebook community. This increases the polarization of those for and against conventional medicine. On the other hand, Glaeser [60] demonstrates that if people have strong previous convictions, then the corrections of false information can backfire by increasing polarization among those participating in discussion. Above all, this is seen to be true in the persistence of the same issues about Di Bella multitherapy, over a period of 20 years, as reported by Passalacqua [54].

The amount of conversations retrieved about nutritional and herbal remedies together comply with the trends in CAM consumption reported by current literature [5,6,15,35,61]. In particular, D’Arena [14] reported that Italian cancer patients resort mostly to aloe formulations, green tea, and supplements. But in our findings, a clear distinction between nutrition and supplements and plant-based medicines emerges. We observed a wide consensus on food and diets effectiveness to face cancer. Conversely, plant-based medicines and supplements benefits are more argued. Moreover, “chemical-based” medicines are more controversial. This complies with CENSIS report [18]: remedies that claim a “natural” background are perceived as more effective and safe than the “chemical-based” ones. Web users tend to consider “nutritional” and “natural” remedies harmless. Conversely, food chemically or industrially processed such as meat, fish, carbohydrates, complex sugars, and milk products are considered too adulterated and harmful.

Despite the wide consumption of some popular CAM therapies reported by the literature among the Italian cancer patients [5,18,62], we collected a very small number of conversations about these topics. Probably, this is due to the fact that acupuncture, phytotherapy, homeopathy, homotoxicology, anthroposophic medicine, ayurvedic medicine, and Chinese traditional medicine are accepted as medical acts by professional bodies of Italian physicians [63], but also to the fact that the “nutritional” issues are currently mostly discussed in other Italian media, such as television (ie, “Le Iene” TV show).

Contrary to the trends observed by the Social Oncology Project [39], the volume of Italian Web conversation is matched with the impact of the disease. In fact, the Italian Association of Cancer Registries [64] reports that breast cancer was the most common incident cancer among women in 2011 (13%) and also the most prevalent neoplasm from 1992 to 2006 (522,235 cases). But the prevalence of gynecological cancer conversation could be related also to some predictors of CAM use and the Internet health information consumption. Molassiotis [5], Jong [16], Yun [59], and Hubner [15] agree that women with chronic disease, high education, and good socioeconomic status are more inclined to use CAM. At the same time, Kelly [65] reported that active health information seeking using a wide range of sources was predicted by female gender, aged 40-64 years, higher education. The trend among Italian people is the same [14,18,66]. Siliquini [66] reported also that women with chronic disease, aged 42-53 years, are more susceptible to negative modification in health behaviors induced by Web information.

### Limitations

Our study has some limitations. The sample is limited in number, and it is not representative of all the Italian Web conversations in the period taken into account. Furthermore, it was not possible to acquire demographic information about Web users. Although we have included a consistent number of Web users, this may not be representative of the entire Italian population. The use of mean and standard deviation for summarizing the opinions collected using a Likert-type ordinal scale is controversial; however, given the adequate sample size of each group and the nearly normal distribution of our data, the parametric approach is considered acceptable [66].

### Conclusions

Conversations on CAM and cancer are ubiquitous. Most of them flow through nonhealth-related Web sources. We need to know people’s information background to better understand their “implicit” knowledge about CAM issues and cancer. We found that unsubstantiated beliefs such as Di Bella multitherapy last for many years, despite scientific evidence. This particularly happens with the cancer patients’ Web-based communities that were retrieved. Understanding the reasons for this persistency against scientific evidence needs further research. There is a big concern about the biologically based therapies and remedies, in particular diet and nutrition and naturopathy issues. These are perceived as harmless and useful, under-rating all risks related to dangerous interactions or malnutrition.

Based on our data, we suggest that doctors should be aware of the implications of these beliefs for the clinical practice. At the same time, it is necessary to keep themselves up to date about the ever-changing CAM issues, to foster patients’ information requests and avoid decisions based only on autonomous Web searches.

We can exploit Web conversation analysis as a strategy to get insights of people perspective and improve new information strategies and resources even for other controversial topics such as clinical trials, sexuality and fertility, and HPV vaccination.

## Appendix

Multimedia Appendix 1 Extended description of the data collection and analysis workflow.

## Abbreviations

CAM: Complementary and alternative medicines
CENSIS: Centro studi investimenti sociali (center for social investment studies)
CRO: Centro di riferimento oncologico Aviano, national cancer institute
MSS: Mean sentiment score
NIH: National institute of health
OP: opinions
SPL: scientific and patients library of the Centro di Riferimento Oncologico Aviano
WD: Web documents
WS: Web sources
