# Supplementary material for: Web Conversations About Complementary and Alternative Medicines and Cancer: Content and Sentiment Analysis
Source: J Med Internet Res. 2016 Jun 16;18(6):e120. doi: 10.2196/jmir.5521 (PMC4929351; doi:10.2196/jmir.5521)
Supplement: Multimedia Appendix 1 [file jmir_v18i6e120_app1.pdf]

Multimedia Appendix 1: Data collection and analysis workflow

Our data collection methodology is similar to theoretical sampling [46], because it involved simultaneously collecting, coding and analyzing textual data from the web conversations. The textual data analyzed in this work have been collected through ifMONITOR [47], an automatic WI (Web Intelligence) tool developed by AILAB (Artificial Intelligence Laboratory of the University of Udine). ifMONITOR provided the following services:

a) *Web filtering*: given a specific research topic of interest, it is possible to define a search profile, to meta-search the Web and filter out any irrelevant results with a programmable combination of search strategies.
b) *Web Monitoring and Thematic Database Construction*: periodic access to previously selected online sources for collecting and filtering all new information available. The relevant information filtered are subsequently stored in a database associated to the specific search profile. The thematic database can be accessed through a specific internal search engine or through tags automatically attached to documents (see pt.c) and it provides data mining tools for analyzing this content.
c) *Automatic Classification*: this service operates through an auto-tagging mechanism, capable to analyze the content of a document and to detect specific patterns or contents present in the text, and subsequently assign to that text a specific tag, corresponding to the pattern discovered

Within our search, we followed a systematic approach, which was organized into six phases, with a strictly functional approach. We have used ifMONITOR for services “a” and “b” in phase 3, and for service “c” in phase 4.

The entire workflow has been summarized in figure 4.

Figure 4. Web conversation analysis workflow

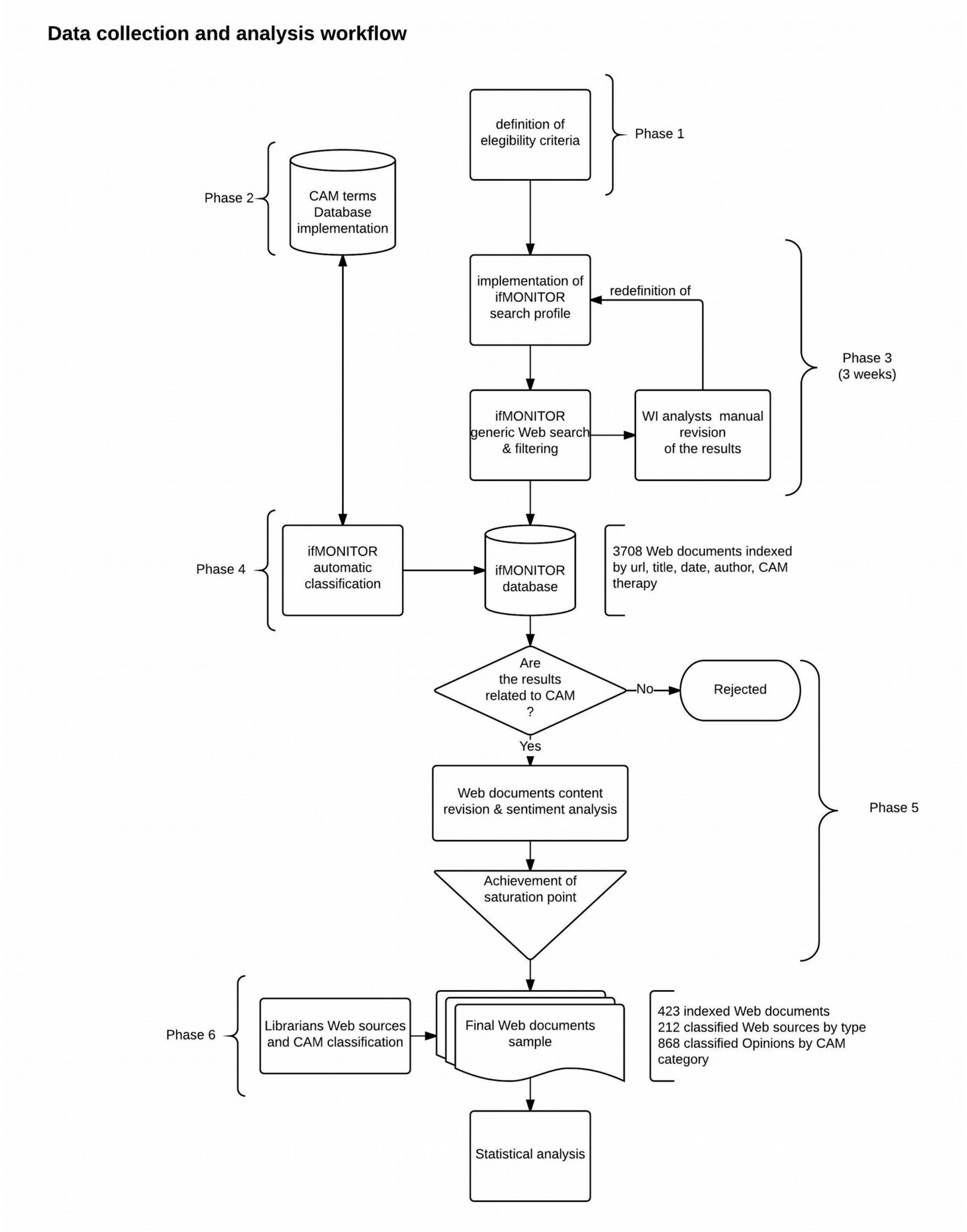

Phase 1: Eligibility criteria definition

Websites, forums, blogs, communities, social networks of any kind and topic addressed to the general public and containing conversations about CAM topics have been included. In particular, Facebook profiles, pages and groups of users and Youtube channels were also included (dependingon the privacy settings). Otherwise we decided to leave out Twitter because the length of the messages is too short to express a complex opinion about the topic taken into account. The web search was also limited to web documents published between January 1 2013 to May 31 2014. We also limited the Web search only to Italian language Web documents, to meet the perspective of Italian people with low literacy skills.

Phase 2: Identification and specification of CAM terminology

Phase 2 is manual. Carried out by the CRO librarians, starting from the general topics to be explored. The first objective was the identification of all the relevant terms to be used as keywords related to cancer and to CAM therapies.

The second objective is to identify a specification process constituted by the development of CAM therapies database, to be used as a reference.

Since the final goal was to analyze online conversations, it was important to capture the terminology which is actually known and commonly employed. For these reason, three main kinds of information sources have been taken into consideration:

- Scientific and medical resources [1,48,49]
- Specific educational booklets for patients published by Cancer Volunteer Associations [50,51]
- Public online sources concerning CAM in Italian language easily accessible on the Web [52,53].

The analysis of such sources was aimed at identifying existing CAM therapies and possible alternative terms used for referring them. Moreover, in order to develop a CAM therapies database, a structured template was filled up, specifically designed for gathering useful information for later phases for each CAM therapy identified. For each therapy or remedy we found a synonyms. References to specific philosophy or religion; names of people, organizations and places; book titles; anatomy and physiology terms were collected when available.

Phase 3: Automatic filtering the web

In this phase all material to be later analyzed exploiting ifMONITOR services “a” and “b” was collected, and it is in three steps:

1. *Identification of a specific basic set of keywords to be used in ifMONITOR*: a set of some generic keywords for search terms was identified to be exploited as search terms. This set included, among others: “cura cancro” (cancer therapy), “rimedi per il cancro” (cancer remedies), “rimedi per neoplasia” (neoplasia remedies), etc. The choice of generic keywords was made for enlarging the number of documents collection and also to simulate queries made by real people accessing the Web. Generic search engines are evidently the preferred tool for finding health information online. Conversely, very few Italian users visit health institutions Websites to obtain information [38].

2. *Launching ifMONITOR*: by means of the initial set of keywords, a specific search profile was initialized within ifMONITOR.
3. *Analysing ifMONITOR's output and re-launching*: in the first period, the reviews of the collected documents were manually performed. These reviews aimed at refining the search profile, extending the set of generic keywords and adding other online resources adequate for gathering documents which may be relevant to our topic of investigation. The Web Monitoring System was launched several times over a period of about ten days, and successively refined and improved search profile on a more focused set of online sources related to people's conversations. At every activation of ifMONITOR, the relevant retrieved documents were stored in the Thematic Database associated with the search profile. For dealing with the polysemy problem, a list of specific adequate filtering rules were inserted in the search profile to filter out non relevant documents (i.e. cancer as a zodiac sign). Another side effect of manual reviews was the discovery of other not previously encountered CAM therapies.

The final result of phase 3 is a set of 3708 documents largely referring to people's conversations which were processed in phase 4.

Phase 4: Automatic classification

This fully automated phase is aimed at classifying a total of 3708 retrieved documents according to which CAM therapy is mentioned in the document. The starting point was the terms included in the CAM Therapies Database. Several documents mentioned more than one CAM therapy and several did not contain any reference to any of the terms included in the CAM Therapy Database. Analogously, two other classification schemes were added to the tagging mechanism provided by ifMONITOR: one for specific cancers and another for identifying the part of human body interested by cancer.

Phase 5: Selection of the final sample and sentiment analysis

This phase is mainly manual. It aimed at identifying a sample of documents to be analyzed in detail. First an automatic filtering out of all documents which had not been tagged in phase 4 was performed on the Thematic Database of 3708 documents: this operation gave 838 documents which were then manually analyzed starting from the most successful online sources (websites, blog, forums publishing a high number of pages on CAM therapies). This criteria allowed to take into consideration the most popular sources and web conversations first.

This process continued up to the accumulation of 423 Web documents, when we reached the saturation of the sample [46]. We considered this to be a significant and feasible size for the sample of documents to be manually analyzed in detail since the remaining Web documents retrieved did not shed any further light to the evaluation of the most relevant Web conversation sources or the mostly mentioned CAM therapies.

For each document, a manual tagging concerning the sentiment about the perceived effectiveness/ineffectiveness of the CAM therapy mentioned through a numerical score ranging from 1 to 5 (1 very bad; 2 bad; 3 neutral; 4 good; 5 very good) was added. The score assignment was performed by a team of four trained WI analyst. They performed the sentiment analysis on different sets of documents individually. Later they compared the score assignments. Thus limiting information bias due to inter-operator discretionary.

We did not perform any assessment of the medical and scientific reliability of these statements.

Phase 6: Final sample classification

According to literature, the librarians tagged the documents included in the final sample using five CAM therapies classes. Within each class, they grouped CAM therapies in 14 categories as shown in Table 1 included in the article.

 Scientific and Patients Library librarians later indexed the Web sources according to the criteria of health and non-health thematic areas. Web sources are Websites, Blog, Forum, Social Networks (personal profiles, groups of users or public pages). Then the librarians gathered Web sources accordingly to their specific subject matters, as shown in Table 2.

The content classification allowed us to identify the most discussed CAM therapies; the most relevant Web sources; the most discussed cancer type. The semantic analysis allowed us to measure popularity and sentiment about the identified CAM and identify the pros & cons issues in the discussions about the most discussed CAM therapies.
